# Are Coastal Hotels Ready for Climate Change? The Case of Alexandria, Egypt

**DOI:** 10.3390/ijerph20065143

**Published:** 2023-03-15

**Authors:** Mahmoud Abou Kamar, Nadir Aliane, Islam Elbestawi, Mohamed Fathy Agina, Omar Alsetoohy

**Affiliations:** 1 Faculty of Tourism and Hotels, University of Sadat City, Sadat City 32897, Egypt; 2 Management Department, College of Business Administration, King Faisal University, Al-Hassa 31982, Saudi Arabia; 3 Hotel Management Department, Higher Institute for Specific Studies, Heliopolis, Cairo 11771, Egypt

**Keywords:** climate change, coastal hotels, vulnerability, remote sensing, GIS, Alexandria, Egypt

## Abstract

Climate change casts a shadow on the tourism industry in Egypt in general, and on coastal hotels in particular, as the coastal areas of Egypt have been classified as the most vulnerable to climate change in the Middle East. As such, mitigating the negative impacts and threats of climate change requires an assessment of the vulnerability of coastal hotels and the extent to which adaptation measures are applied. Accordingly, this study applied a hybrid methodology to achieve three main objectives. First, to evaluate Alexandria’s vulnerability to future climate change (at the destination level) by analyzing the recent climatic trends and expected scenarios. Second, to assess the vulnerability of Alexandria’s coastal hotels to climate change (sector level) using satellite images, aerial mapping, remote sensing, and geographic information systems (GIS). Third, to explore how coastal hotels are adapting to the risks of climate change using the four business-focused adaptation measures (i.e., technical, managerial, policies, and awareness-raising). The findings of the study revealed and confirmed that the hotel sector in Alexandria is threatened by sea level rise (SLR). Four hotels are at risk of inundation, and the extent of hotels at risk will increase with future scenarios of SLR. On the other hand, the results of examining the adaptation measures of 36 hotels indicated that the scope of the adaptation measures differed significantly between hotels due to factors such as hotel category, size, duration of operation, and EMS status, but overall, the scope of application was more comprehensive and varied than expected. Technical adaptation measures were the most common and applied by the majority of hotels in Alexandria. The results of this study should help figure out what adaptation measures coastal hotels should take and show policymakers where they should focus their adaptation efforts.

## 1. Introduction

Egypt’s Mediterranean coast, specifically Alexandria, occupies great importance in the country’s plans for economic development and coastal tourism. This is due to the large number of visitors to Alexandria (2.9 million visitors) and the average stay is projected to be 1.6 nights, while the average stay for European visitors is 1.4 nights and the average stay for African visitors is 2.3 nights [1]. Additionally, coastal tourism is the driving force for sustainable development in Egypt [2]. However, climate change is casting a shadow over Egypt’s tourism industry, with the country ranked as highly vulnerable to climate change in the Middle East [3]. Disturbingly, climate change takes the form of an increase in global temperatures that causes sea level rise. As a result, low-lying coastal areas including but not limited to those dependent on coastal tourism are at imminent risk [4]. The term “*coastal tourism*” refers to any type of touristic, recreational, or amusement-based activity that takes place along or near the coast. Hotels, restaurants, the food business, and second residences are all examples of coastal tourism developments, as are infrastructures such as shopping centers, marinas, and service providers [5]. The economic significance of coastal tourism is unquestionable. In the last few decades, it has emerged as the fastest-evolving industry in many countries [6]. However, coastal tourism is one of the economic sectors that are most susceptible to damage as a result of the potential climate change. This is because coastal tourism is intimately connected to natural and environmental resources such as the weather, beaches, and sea, all of which are regarded as being extremely sensitive to climate change. Indeed, the World Tourism Organization (WTO) considers climate change as the most substantial challenge to tourism’s development potential [7]. Climate change has severe impacts on coastal tourism activities in terms of changing the spatiotemporal distribution of temperatures, the receding of drinking water, droughts, rising sea levels, property damage [8], the availability of the beachfront, and the quality of the coastal territory [2]. Such effects have been evaluated on coastal areas in Egypt, especially the Mediterranean coast, which stretches for about 1200 km. According to the World Bank [9], 6.1 million coastal residents might be displaced and 4500 km^2^ of agriculture would be destroyed, resulting in an Egyptian GDP loss of 6% for a 1 m sea level rise and 16% for a 5 m sea level rise. Climate change, according to Refaat and Eldeberky [10], will have an impact on Egypt’s coastal infrastructure and inhabitants unless mitigation and adaptation policies are put in place. Low-lying regions such as agricultural and urban areas will be wiped out by rising sea levels. Climate change will impact not only the physical and ecological features of coastal regions, but also the social fabric of such areas [8]. According to Scott et al. [11], adaptation strategies are required to mitigate the impact of these changes on coastal tourism.

Not surprisingly, such challenges have a direct impact on coastal hotels in developing countries [5] including Egypt. On one hand, these countries need to sustain the social and economic returns they derive from tourism. On the other hand, they need to address their shortcomings and limited adaptation potential. Despite these significant and far-reaching challenges, there remains a paucity of studies exploring the readiness of coastal hotels for the impact of climate change and sea level rise [12]. Furthermore, only limited empirical studies have been conducted to determine the impact of climate change on tourism on the African continent [13].

The coastal hotel sector has not been scrutinized to determine the adaptation strategies applied by this sector. Until recently, the hotel business devoted little attention to environmental concerns and climate change [14,15]. It is particularly pertinent for coastal hotels to anticipate future climate changes, elicit their consequences, and build more robust and sustainable adaptation strategies [16]. Academics believe that adaptive strategies should be prioritized to prepare businesses for impending challenges. This tendency is consistent with the declaration published under the United Nations Framework Convention on Climate Change, which urged governments to pay particular attention to climate change adaptation strategies [5]. Not only do adaptation strategies help organizations make important decisions about how to deal with climate change scenarios, but they also help them to be better able to lessen the negative effects of a recurring event [8].

Given their prominent role as tourist facilities and income generators [15], hotels should be a major focus of coastal adaptation studies. Accordingly, it is not surprising that the 13th Sustainable Development Goal (SDG), “Climate Action”, proclaimed by the International Council for Science [17] emphasizes the importance of developing effective adaptive strategies to mitigate the negative impacts of climate change in developing countries. Given the importance of hotels in bringing about economic and social development in coastal areas, it is of paramount importance to understand how coastal hotels in Egypt strategically plan to adapt to confront the threats of climate change. In the academic context, both [18,19] stated that there was a lack of studies on the implications of climate change on the hotel industry, particularly in developing countries [11,20]. Thus, assessing the readiness of coastal hotels in Egypt to adapt to climatic changes is a critical issue at the national and regional levels. To fill this knowledge gap, this study focused on the state of Alexandria to achieve the following objectives:

1. Evaluate Alexandria’s vulnerability to future climate change (at the destination level) by analyzing the recent climatic trends and expected scenarios that are supported by scientific evidence.
2. Assess the vulnerability of Alexandria’s coastal hotels to projected climate change (sector level) using satellite images, aerial mapping, remote sensing, and geographic information systems (GIS).
3. Explore how coastal hotels in Alexandria are adapting to the impacts of climate change.

## 2. Theoretical Framework

### 2.1. Climate Change and Adaptation Strategies

Climate change is defined as an imbalance in the usual climatic conditions such as temperature, wind patterns, and precipitation, which characterize each region on Earth. Climate change, according to the Intergovernmental Panel on Climate Change [21], is defined as detectable changes in the climate, based on statistical analysis, and lasts for decades or even more, whether caused by natural oscillation or as a consequence of human activities. The frequency and magnitude of long-term global climatic changes will have massive consequences for natural vital systems. Increasing temperatures will also lead to a change in weather types such as wind patterns and the amount and types of precipitation, in addition to the occurrence of several potential extreme weather events. This will lead to wide-ranging and unpredictable environmental, social, and economic consequences [22]. In a recent revelation, the National Aeronautics and Space Administration (NASA) stated that the continual shift occurring in the climate was having an impact on the ecosystem and was paving the way for a new geologic age, which some refer to as the *Anthropocene*, in which the climate will be substantially different from the one our forefathers knew [23].

When examining the expected effects of climate change, two types of impacts can be distinguished. The first category includes effects that will not be felt for a very long time such as rising sea levels and temperature, while the second covers other effects such as severe rainfall, drought, saltwater intrusion, and an increase in storms, which exacerbate the sediment instability conditions inside the coastal zone and produce further coastal erosion [24]. These severe effects are uncommon, but their ramifications can be highly disruptive and destructive [8]. As a result of the various types of climate change consequences, severities, spans of vulnerability, and chasms of effect, tourism businesses must consider a broad spectrum of adaptation actions. The Intergovernmental Panel on Climate Change (IPCC) defined climate change adaptation as “*the process of adjusting to the existing or predicted climate and its impacts*” [21] (p. 118). More specifically, the UNFCCC (United Nations Framework Convention on Climate Change) embodied adaptation as modifications to environmental, cultural, or economic systems in response to present or anticipated climatic impulses and their consequences or implications. In its simplest form, it entails applying processes, activities, plans, and technologies to mitigate potential disruption or capitalize on advantages associated with climate change [25] Subsequently, tourism operators (including hotels) must prioritize climate change adaptation and implement relevant adaptation measures [14,26]. Previous exploratory studies have investigated the susceptibility of coastal tourism to the effects of climate change as well as adaptation strategies. For instance, Santos-Lacueva et al. [27] investigated how decisions, policies, and strategies affect a coastline’s climate change vulnerability. Empirically, Scott et al. [16] conducted empirical research on the vulnerability of Caribbean resorts to climate change. According to their findings, if sea levels rise by one meter, 29 percent of resorts will be flooded. Other studies have either focused only on the conceptual aspects of this issue [27], or have focused on adaptive culture [28]. According to Nalau and Leal Filho [29], adaptation to climate change is influenced by different factors that include cultural, socioeconomic, historical, and environmental contexts.

For instance, Becken [30] assessed the mitigation and adaptation practices of Fiji’s coastal resorts. The study initially categorized the effects experienced by hotel managers into either structural or behavioral measures. Structural adaptation measures included infrastructure structural integrity, emissions to the atmosphere and air quality, energy and water savings, hazardous materials management, waste management, noise reduction, and pollutant reduction. While the behavioral adaptation measures included increasing guest awareness, the protection of marine creatures, emergency evacuation plans, and indoor activities, the study concluded that structural measures were more significant and applied more frequently than behavioral measures. Another Pacific study conducted by Parsons et al. [28] investigated the Pacific Island country of Samoa’s adaptation potential within its unique setting and culture.

In turn, Weaver [31] argued that adaptation to climate change is a logical response that is not directly related to environmental, social, or cultural sustainability. He also added that tourist establishments including hotels must adopt mitigation strategies to achieve real and tangible benefits in the short- and medium-term. Correspondingly, local sustainability issues such as air quality and biodiversity protection must be addressed. In response to [31], Barnett et al. [32] argued that adaptation strategies to climate change are constrained by three main dimensions: physical environmental, technological, and economic aspects. For example, due to the complexity associated with ensuring long-term sustainable social adaptation, it is uncertain how some environmental or physical restrictions may be overcome. Additionally, the current technology’s adaptability to climate change is limited [33].

In Africa, there is an expanding corpus of studies on climate change adaptation and mitigation strategies for the tourism industry. For example, in a study on vulnerabilities and adaptation to climate change in South Africa, Hambera [34] emphasized the nonchalance toward climate change by tourism operators and the government. Additionally, Hoogendoorn and Fitchett [35] emphasized the urgent need for more studies in African countries regarding the threats of climate change to the African tourism sector, noting that such research has so far only been directed to developed countries. Mahadew and Appadoo [36] assessed the adaptation of tourism facilities in Mauritius to climate change based on the UNEP Framework on Tourism and Climate Change. As a result, the long-term goals cannot be reached because the measures are not goal-oriented or regulated, which makes them impossible. In the context of Egypt, Sharaan et al. [37] reviewed Egypt’s efforts, tactics, and experiences in combating coastal erosion, floods, and sea level rise resulting from floods. Egypt’s national policy for adapting to changes on the coast was to build seawalls, berms, artificial dunes, and sand mats made of natural plants to trap sand. Several defensive initiatives such as the development of new fish farming techniques, the routine draining of coastal lakes and lagoons, and the maintenance of the coastal road were also identified. Over the past few years, many of these adaptation strategies have proven effective in preventing damage to the coastline caused by rising sea levels.

### 2.2. Climate Change and the Hotel Sector

Although studies on the relationship between tourism and climate began in the 1960s, actual interest in this topic did not begin until the 1990s, when researchers took a more serious look at the phenomenon of climate change and its effects on tourism [38]. Currently, the field is “mature” enough to attract the interest of governments, policymakers, hotels, and other tourism establishments [39]. Climate change and tourism have a bidirectional relationship so that tourism activities (including hotels) are both influenced by and key contributors to this issue [38]. According to Frey and George [40], only 2% of tourism activities are engaged in sustainable activities in response to the issue of climate change.

**Figure 1:**
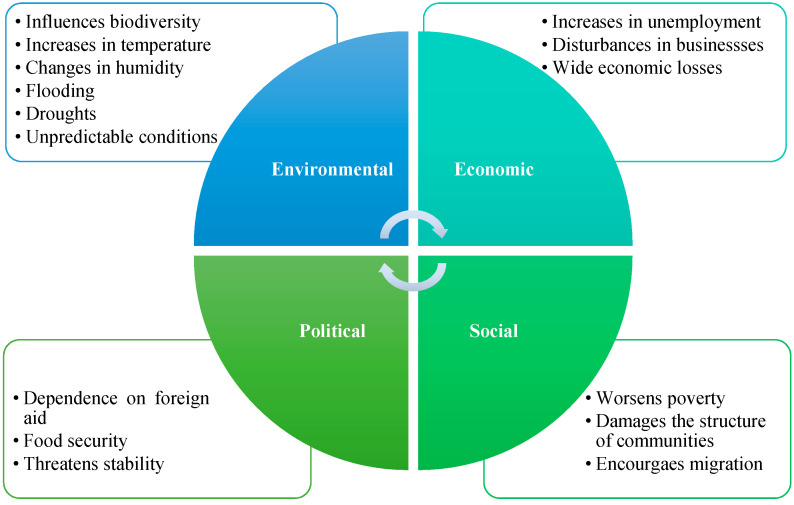
The intrinsic impacts of climate change on the tourism industry. Source: Adapted from [44].

While tourism is not a significant contributor to carbon dioxide emissions when compared to other industries, its emissions are predicted to increase rapidly and will affect its contribution levels [41]. According to WTO estimates, tourism has exacerbated global climate change by causing the emergence of nearly 5 percent of carbon dioxide emissions [42]. In 2008, tourism’s impact on climate change was estimated to be between 5% and 14% of the global warming caused by human greenhouse gases [42]. From 2009 to 2013, worldwide tourism’s carbon footprint rose annually, rising to account for almost 8% of global greenhouse emissions [25]. Simultaneously, tourism is directly and indirectly affected by the repercussions of climate change such as heatwaves and drought, floods, hurricanes, rising sea and ocean water levels, biodiversity disruption, environmental damage, and low destination attractiveness [43]. Figure 1 depicts an outline of the intrinsic impacts of climate change on the tourism industry.

The hotel business is no different from the rest of the tourism industry. Energy-intensive use makes this sector not only the most susceptible to the effects of climate change because of the substantial investments made in physical infrastructure [42], but also the greatest contributor to carbon emissions [42]. Hotels utilize an excessive amount of energy, water, food, wood, and polymers. Many of these wastes require special dumping [45]. Furthermore, hotels generate a variety of unfavorable emissions including CO_2_, which accounts for around 21% of the overall tourist industry emissions [42], chlorofluorocarbons (CFCs), noise, smoking, and odor. Because of the fast evolution of hotels and their energy-intensive nature, the hotel sector is expected to account for almost one-quarter of the tourism industry’s carbon dioxide emissions. Hotel carbon emissions are expected to rise at a 3.2 percent annual rate, reaching 728 Mt CO_2_ by 2035 [46]. As a direct result of this, hotels are required to reduce their emissions of greenhouse gases by 66% by the year 2030 and by 90% by the year 2050 [47]. Understandably, climate change is expected to have negative consequences on the performance and competitiveness of hotels [48]. Table 1 encapsulates a broad range of the negative consequences of climate change on the productivity and profitability of hotels.

It is challenging for the hotel industry to respond to climate change due to the industry’s structure, which involves a high level of human engagement in operations and management as well as transparency concerning various stakeholder groups [26]. Despite these caveats, the majority of studies on the impact of climate change on the hotel industry has been based on the framework of corporate social responsibility. This field investigates the roles and responsibilities of corporations concerning the environment and society. Recently, a growing number of prominent hotel companies have been pledging their support for sustainable development practices to combat climate change to assert their competitive advantage, create their brands, and differentiate themselves from their rivals in the market. Marriott International, for example, has pledged to reduce its carbon footprint to zero by the year 2050 by committing to a goal of net-zero value chain greenhouse gas emissions [49].

Hotels often adopt initiatives to enhance positive impacts and reduce negative outcomes. These initiatives are based on environmentally friendly and sustainable practices, which allow hotels to distinguish themselves from rivals and appeal to environmentally conscious visitors. Examples of these initiatives include increasing environmental awareness in host communities [50], green hotel development [51], the adoption of environmentally friendly practices [52], environmental marketing strategies, and environmental management practices [53]. For example, some studies have focused on issues such as the determinants of energy use in hotels [50]; the use of renewable energy sources [54]; the assessment of carbon and greenhouse gas emissions [54,55]; energy-saving initiatives [56,57]; industry response to climate change [5]; and climate change implications for hotels [58].

The hotel industry, according to Mak and Chang [59], has emphasized the need to adopt strategies for environmental sustainability as a way to improve environmental performance and reduce negative impacts. Specifically, four basic strategies have been proposed: an integrated environmental management system, the adoption of environmentally friendly marketing strategies, the implementation of low-cost environmental strategies, and the pursuit of green certification. Because each of these strategies serves a specific purpose, it is difficult to figure out which one is best. This is because many factors come into play including the hotel’s interests and resources, the influence of stakeholders, and the policies and laws of the countries in which these hotels operate [49]. Within this framework, researchers have developed several climate change adaptation measures [40,60,61,62] to deepen the understanding and taxonomy of adaptation strategies. Table 2 is a summary of the many technical, managerial, policy, educational, and behavioral measures to respond to climate change threats. These measures collectively act to adapt to a changing climate. In the absence of an appropriate adaptation framework for the tourism and hotel sector that is widely accepted and universally applicable, the current study adopted the framework proposed by Simpson et al. [42], as it is more suitable for the tourism and hotel sector and can be applied in the coastal hotel business environment in Egypt.

### 2.3. Alexandria: The Study Area

**Figure 2:**
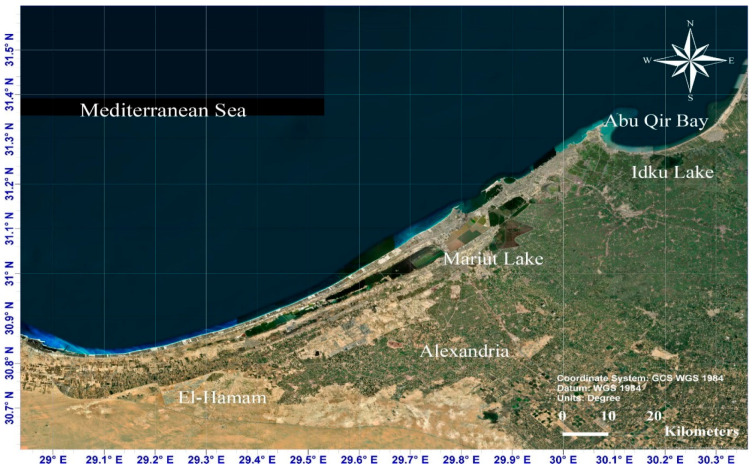
Satellite image of the Alexandria coast.

Egypt has a coastline of roughly 3500 km, with about 1200 km on the Mediterranean and about 2300 km on the Red Sea. These coastlines are at risk from sea level rise and floods brought on by extreme climate change. The Egyptian Mediterranean coastline, from Rafah in the east to Saloum in the west, encompasses key cities such as Alexandria, which is located on Egypt’s north coast. Alexandria’s coordinates are approximately 30°50′–31°40′ N, and 29°40′–32°35′ E. It has an uneven topography in the south, with hills between 0 and 40 m above sea level, and a gradual slope into the Mediterranean Sea in the north [63]. Alexandria is home to the second-largest metro area in Egypt and is a prominent city in the Mediterranean region, with a population of over 5.3 million people (or about 5.5% of Egypt’s total population) and a population density of 3044/km^2^ [64]. Nearly 40% of Egypt’s manufacturing takes place in Alexandria, which also possesses the country’s largest harbor, serving over 60% of Egypt’s imports and exports [65]. Furthermore, it has significant investments in the industrial, tourism, and agricultural sectors. In addition to its importance as a tourist destination, the coastal area of Alexandria is a major source of fishing, accounting for more than 13.3 percent of Egypt’s total fish production [65]. Along Alexandria’s coastal strip, hotels, yacht docks, seaside facilities, and infrastructure have been constructed. These facilities and the related tourism activities mostly draw tourists from the surrounding area as well as tourists from neighboring Arab countries, and a limited number of international tourists [2]. During fieldwork in 2021 and 2022, there were forty-three hotels in Alexandria, comprising 4300 rooms, representing about 55% of the total hotel capacity on the northern coast of Egypt [66]. Because of its relatively low elevation (see Figure 2), the Alexandria coastline is vulnerable to submergence risk [67]. Using historical data, Sharaan and Udo [68] projected the potential retreat and loss of coastline along the northern coast of Egypt in the year 2100 including the coast of Alexandria. Abu Qir Beach in Alexandria is believed to be the most exposed to severe dips and erosion along the northern coast of Egypt.

The data in Table 3 reflect the findings for the predicted coastline retreat by meter, the analogous shoreline erosion in meters per year, and the beach loss (percent) for the various sea level rise scenarios. In 2100, sea flooding is anticipated to force the exodus of 6.5 million Alexandrians [69].

**Figure 3:**
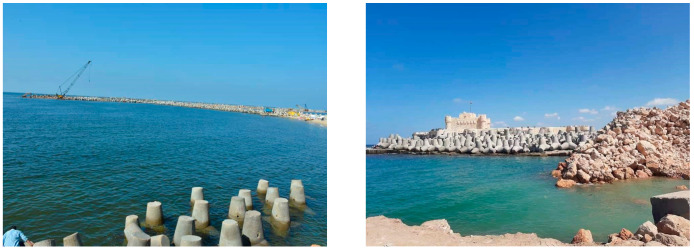
Seawalls, submersible barriers, and soil stabilization to protect the coast of Alexandria.

The Alexandria Research Center for Adaptation to Climate Change has revealed that the adaptation responses to deal with sea level rise include conducting extensive studies to determine the efficacy of the proposed adaptation measures, developing additional regulations for coastal development such as Environmental Impact Assessments (EIAs), and establishing artificial nourishment with sand to compensate for beach erosion may be accompanied by the establishment of solid protection measures such as stone heads or submerged barriers, if necessary [70] (see Figure 3).

## 3. Hybrid Research Methods

The data for the current study were derived from a combination of different sources (basically qualitative and quantitative). First, to assess the vulnerability of Alexandria’s coastal hotels to projected climate change and projected sea level rise (SLR), many materials including image data and processing satellite images from [71] were processed to obtain the current shoreline of Alexandria. To delineate the shorelines, images were downloaded from the USGS website and geometrically adjusted to conform to the Universal Transverse Mercator (UTM) format, Zone 35 North, and WGS84 data. We employed the Shuttle Radar Topography Mission (SRTM) digital elevation model (DEM) to estimate the elevations of the study area and create the expected scenarios of the increase in sea level in accordance with RCPs as well as SSP5 and SLR 2m, respectively. From the STRM data, a DEM of Alexandria was created to detect the study area elevations. A DEM is a digital representation of the terrain on the Earth’s surface and is an essential component in hydrological models [72] (see Figure 2).

According to Amin and El-Fatraiy [73], SRTM and local DEMs have almost the same accuracy in terms of the root mean square error (RMSE), whereas Advanced Spaceborne Thermal Emission and Reflection Radiometer ASTER DEMs rank lower. The accuracy of ASTER DEMs was significantly improved (49.5%) once vertical shifts vs. GCPs were eliminated. The omitted values were a systematic shift; therefore, the model is a relative DEM. SRTM DEM may be used to update 1:50,000 scale topographic maps across flat and steep terrain since its RMSE is less than half the contour interval. ASTER DEMs can be used to update lower-scale topographic maps. The spatial resolution of the SRTM data that were utilized was three arc seconds (approximately 90 m at the equator). The representative concentration pathways (RCPs) and shared socioeconomic pathways served as the basis for our four SLR scenarios (SSPs). According to Table 4, we used the mean estimates of RCP8.5 for the years 2046–2100, SSP5 until the year 2150, and the SLR 2m scenarios.

Second, to explore how hotels are adapting to the consequences of climate change, an online survey was administered to the general managers of all hotels in Alexandria. This technique was ideal in light of the exceptional circumstances imposed by the COVID-19 outbreak. The list of hotels was determined based on the data recorded in the 2021 edition of the Egyptian Hotels Directory, which is annually issued by the Egyptian Hotel Association (EHA). The list of hotels included a total of 43 hotels. To make sure there were enough responses, all of the hotels in the directory were asked to fill out the online survey. Definitions of climate change and climate change adaptation were given in the pre-survey emails to give the respondents a frame of reference for their responses. The survey focused on the best strategies to adapt to and mitigate climate change. The Simpson et al. [42] adaptation framework was utilized to categorize the adaptation measures into technical, managerial, policies, and education-focused adaptation measures. The survey was sent out on 21 February 2022. Follow-up calls were made two weeks after the survey had been sent out. In some cases, hotels claimed that they had not received the link to the survey form, so the survey was resent by email. After a further week, the same process was carried out. Thirty-six hotels responded to the survey when it was sent out. A comprehensive analysis of the adaptation efforts of the 36 hotels was carried out using key hotel characteristics (i.e., hotel’s category, duration of operation, size, and status of implementing an environmental management system) (see Table 5).

## 4. Findings and Discussions

### 4.1. The Vulnerability of Alexandria’s Coastal Hotels to Projected Climate Change

Alexandria sits on a T-shaped peninsula that is wedged by the Mediterranean Sea, a series of lagoons, and the remnants of many lakes. Flooding and poor drainage are significant issues since large parts of the city are below sea level. The city is particularly susceptible to ecological risks due to its geographical location and urban layout. The World Bank predicts that by 2030, the city will be much more at risk from factors such as marine submersion, sea level rise, coastline erosion, earthquakes, floods, and a lack of fresh water [74]. Sandy shores line the Alexandria coast, with rocky outcrops serving as natural breakwaters. The combination of persistent natural coastal processes and sediment scarcity results in the gradual but chronic long-term erosion of beaches at a rate of roughly 20 cm per year. Since the seaward enlargement of the Corniche Highway (1998–2002), more than half of the sandy beaches between Montazah and El Silcila, a distance of 14.5 km, have considerably vanished, leaving behind “sediment-deprived” coastal cells. The erosion risk map for 2030 shows that the risk is greatest on the periphery.

Sea level rise (SLR) is a consequence of global warming, which causes the oceans to warm and expand, and the melting of glaciers and ice sheets. The rate of sea level rise depends on several factors including greenhouse gas emissions, ocean circulation patterns, and the response of ice sheets to warming temperatures.

**Figure 4:**
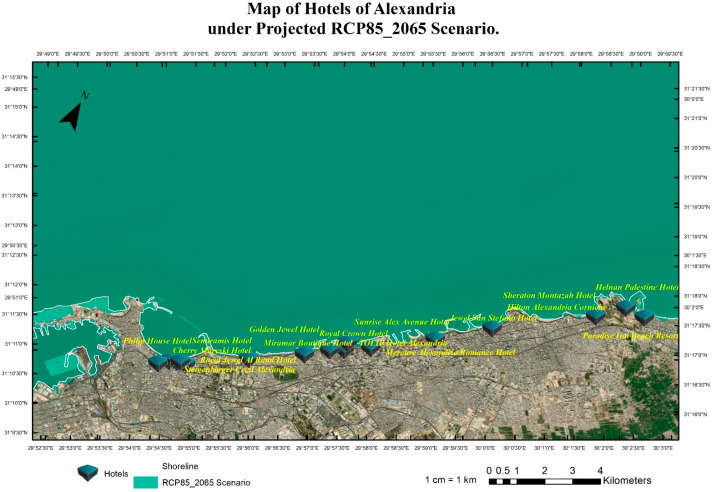
Coastal hotels in Alexandria under the projected SLR scenario of RCP8.5 until the year 2065.

**Figure 5:**
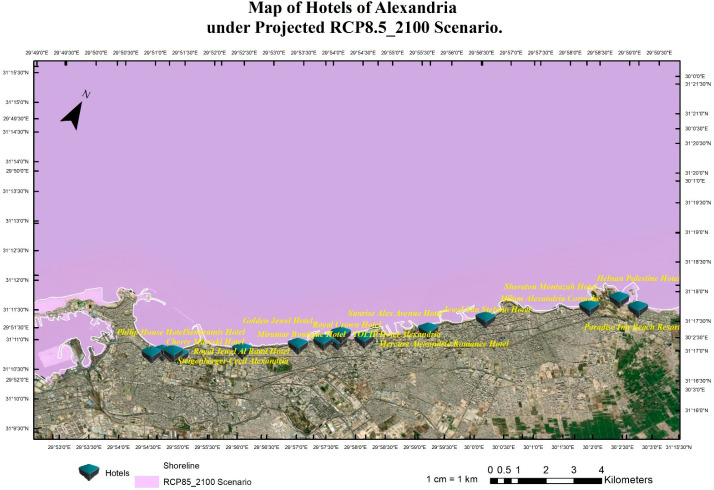
Coastal hotels in Alexandria under the projected RCP8.5 scenario until the year 2100.

**Figure 6:**
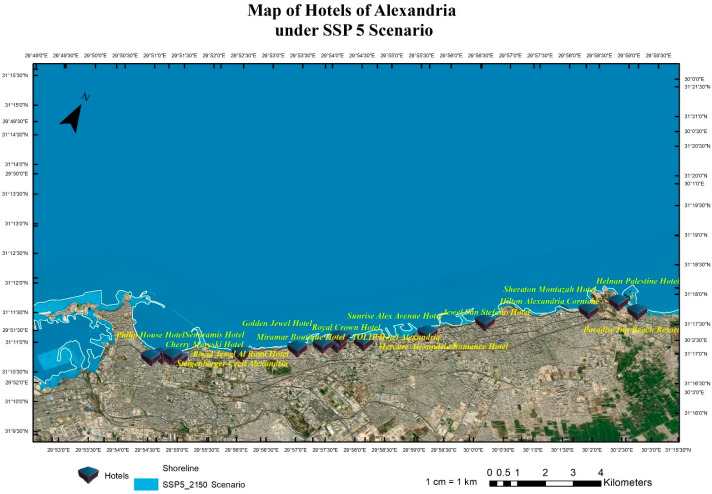
Coastal hotels in Alexandria under the projected SLR scenario of SSP5 until the year 2150.

**Figure 7:**
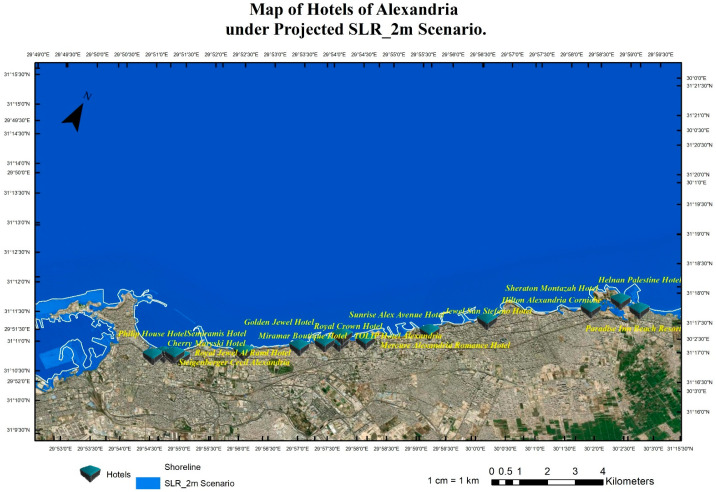
Coastal hotels in Alexandria under the projected SLR 2m scenario.

The research team implemented pessimistic scenarios for SLR because of the sensitivity of tourism buildings and facilities to SLR impacts that will cause quick coastal erosion. Four scenarios were implemented: RCP 8.5 (the year 2065), see Figure 4, RCP 8.5 (the year 2100), see Figure 5, SSP5 (the year 2150), see Figure 6, and SLR 2 Meter (Figure 7). The Representative Concentration Pathway (RCP) 8.5 is a high-emissions scenario that assumes continued increases in greenhouse gas emissions through the 21st century. By the year 2065, under this scenario, it is projected that global sea levels could rise by about 0.22 to 0.38 m (with a mean of 0.30 m) (IPCC, 2013). By 2150, SLR could reach 0.45 to 0.82 m (with a mean of 0.63 m) (IPCC, 2013). These projections indicate that under RCP 8.5, sea level rise will continue to accelerate through the 21st century, with profound implications for coastal communities, infrastructure, ecosystems and coastal tourism facilities, and cultural heritage sites. This scenario represents a future that is characterized by high levels of greenhouse gas emissions and limited action to mitigate the impacts of climate change.

The shared socioeconomic pathway (SSP) 5 is a scenario that assumes high levels of economic growth and energy demand, leading to continued greenhouse gas emissions throughout the 21st century. By the year 2100, under this scenario, it is projected that global sea levels could rise by about 0.98–1.88 m (with a mean of 1.32 m) (IPCC, 2021). Compared to RCP 8.5, SSP5 represents a future that is characterized by a more positive outlook for economic growth but with little progress made in mitigating the impacts of climate change. Although the rate of sea level rise may be slightly slower under SSP5 than RCP 8.5, the impacts of SLR on coastal communities, infrastructure, and ecosystems will still be significant. Using the geospatial analysis of the above-mentioned four different SLR scenarios, these analyses produced the mapping for the hotels in the coast of Alexandria and show the levels of flooding and coastal erosion under different scenarios as shown in Figure 4, Figure 5, Figure 6 and Figure 7.

**Figure 8:**
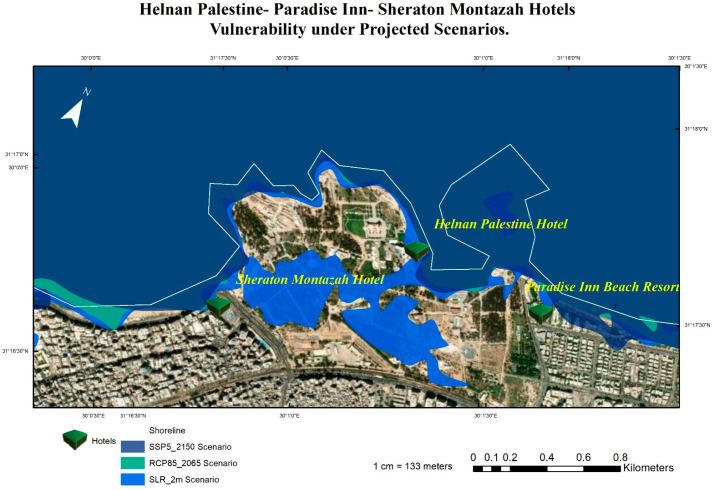
The vulnerability of the Helnan Palestine, Paradise Inn, and Sheraton Montazah hotels under the four projected SLR scenarios.

**Figure 9:**
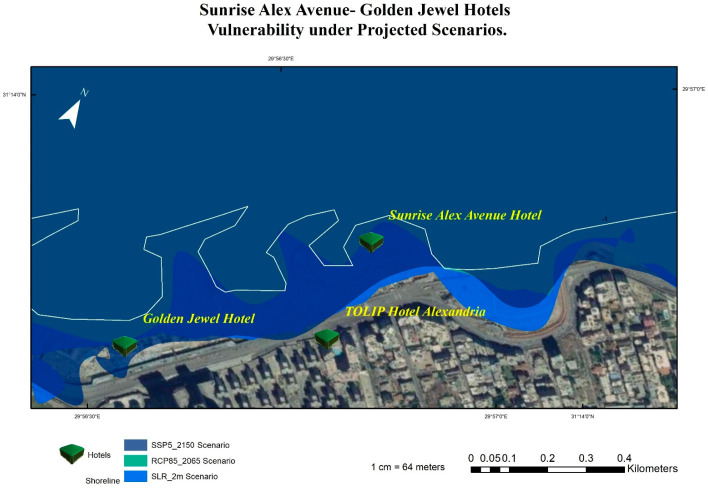
The vulnerability of the Sunrise Avenue, Mercure Alexandria, and Golden Jewel hotels under the projected four scenarios.

According to the results, the most vulnerable hotels under the four SLR scenarios are the Sunrise Alex Avenue Hotel, Helnan Palestine Hotel, Golden Jewel Hotel, Sheraton Montazah Hotel, Mercure Alexandria Romance Hotel, and Paradise Inn Beach Resort. As shown in Figure 8 and Figure 9, the map shows the vulnerability of these hotels under different SLR scenarios.

### 4.2. Climate Change Adaptation Measures by Hotels in Alexandria

This section primarily seeks to explore how coastal hotels strategically plan to adapt to the risks of climate change. The four business-focused adaptation measures (i.e., technical, managerial, policies, and awareness-raising) proposed by Simpson et al. [42] were examined. Cronbach’s alpha (Cronbach’s α) was used to ensure the internal consistency. The overall value was 0.815, while the acceptable value according to [75] is 7. All scales had values greater than 7. Additionally, according to the metadata given in Table 6, all scales were placed within the high degree of application range. This indicates that respondents recognized all measures as significant. However, the relative value of each scale varied from scale to scale. The four adaptive measures with the highest mean values, respectively, were as follows: technical (4.04); policies (3.99); managerial measures (3.88); and awareness-raising (3.53).

The results in Table 6 show that hotels have used a variety of adaptive measures, from simple ones that all hotels can adopt since they do not consume extensive resources to execute, to more complicated and resource-intensive measures. These results are in stark contrast to the findings of previous studies that have indicated the low adaptive capacities of hotels [5], although the general economic conditions in Egypt are, of course, a significant limitation. Particularly striking in the context of a developing country like Egypt is that hotel adaptation measures range from resource-intensive to easy-to-adopt measures. The majority of hotels in Alexandria, Egypt, resorted to technical adaptation measures, even though they usually require large financial and human investments. The most prominent were improving the hotel building insulation and natural ventilation to protect against high heat waves, provide cooling, and thus save energy needed for air conditioning. The design of the hotel reduces the amount of energy needed for cooling, heating, ventilation, and electricity by using high-efficiency windows; double insulation in walls, ceilings, and floors; using less electricity for lighting; making better use of natural light, and using windows or skylights that can be closed or opened to let hot air out during hot weather. This reduces the need for cooling in the summer and heating in the winter. Additionally, making water use more efficient and building more water storage, improving how water is used, especially in kitchens, and promoting technologies that treat and reuse recycled water. However, under technical adaptation, there are other measures highlighted that need substantially fewer resources such as expanding the drainage systems to adapt to heavy rainfall during winter, reducing the energy consumption for cooling, heating, and ventilation, and reinforcing beaches or coastal areas using natural or artificial barriers.

Complying with the national environmental laws and regulations (i.e., managing hazardous substances, wastewater, and solid waste management) was the second reported form of adaptation measures. Although ranked in second place, the potential impact of these measures could be greater due to the extension of their scope to include not only the coastal areas, but the rest of the country.

In third place, managerial measures were the most commonly mentioned adaptation strategy. Hotels can incorporate short- and long-term adaption approaches and initiatives into their overall business strategy as well as warning visitors to avoid potentially dangerous attractions before, during, and after extreme weather events. Despite its importance, awareness-raising came last, with a focus on educating staff and the community on climate change implications and adaptation and potential climate change risks.

For further analysis, an analytical comparison of the results was made based on the main characteristics of the investigated hotels (e.g., hotel category, size, duration of operation, and EMS status) (Table 7). As expected, the extent and scope of adopting adaptive measures varied according to the main characteristics of the investigated hotels. These differences are likely due to the different financial, technical, human, and technological capabilities of the hotels. For example, large hotels (>100 rooms) are at the forefront of the race to implement climate change adaptation measures over smaller hotels. Specifically, large hotels have been more committed and active in the areas of technical, managerial, and policy formulation measures. These results are not surprising as the results of some previous studies such as [5,76] concluded that larger hotels have better environmental practices than smaller ones. When compared to smaller hotels, larger ones engage in a wider variety of endeavors, run more extensive operations, and have a greater societal influence, making them the focus of more scrutiny [77]. They deal with more stakeholders who are worried about the firm’s practices and have higher standards for how the organization handles climate change [78]. Consequently, large hotels face more stakeholder demands, and as a consequence, they are expected and driven to deliver high-quality voluntary adoption measures in response to the needs of the stakeholders [79]. It has also been said that bigger hotels can develop technical measures because they have more resources at their disposal [77,80,81]. Furthermore, one of the key characteristics in this context is a hotel star rating, as it has been stated that higher-rated hotels often have a stronger tendency than lower-rated hotels to apply climate change measures and established environmental practices [82].

Overall, five and four-star hotels have invested extensively in effective adaptation initiatives aimed at minimizing the negative impacts of rising rainfall, sea level, and temperature. The hotel industry needs to ensure positive customer attitudes toward the brand as strategic positioning and differentiation depend on factors other than price and quality alone, and we argue that five-star hotels are always under pressure to meet the customers’ demands or risk losing some of these customers. Consequently, customers often put pressure on large hotels to participate in environmental practices and initiatives. Additionally, it has been found that upscale hotels allocate resources to sustainability management and environmental reporting due to the increased scrutiny of these establishments’ social responsibility initiatives and increased stakeholder pressure [83]. Previous studies have also shown that upscale hotels have an incentive to publicize their green initiatives to attract and retain customers as well as increase their brand popularity and profitability through positive word of mouth and other positive recommendations [84,85]. Therefore, five-star hotels are more likely to adapt to climate change than their less upscale counterparts because their customers are more discerning.

Interestingly, when the duration of time a hotel had been in operation was taken into account, the study results agreed with those of [5], which revealed that hotels with short-term operations were more amenable to adopting environmental concepts and trends including climate change measures. In the same way, this study found that hotels that had been open for three to ten years were more likely to use adaptation measures than hotels that had been open for more than ten years. Finally, the hotel’s use of green systems and practices is a key part of adopting climate change measures. Most of the investigated hotels focused on waste management and energy-saving practices. As for the percentage of hotels that have implemented or intend to implement environmental policies in the future, this was (28%) of the total hotels. This situation is probably because the environmental strategies of many developing countries including Egypt do not force hotel establishments to implement environmental policies. For example, the policy of low water prices, which was meant to help development, has made people less aware of how important it is to keep this resource safe, even though many African countries are in the middle of a water crisis.

## 5. Conclusions

Although the effects of climate change on coastal tourism have been addressed in several recent studies [86,87], there is still a lack of studies focusing on potential climate impacts on coastal hotels [16,88], especially in developing countries with relatively limited adaptive capacities [89]. Tourism businesses in Alexandria are very vulnerable to the potential risks of climate change such as flooding, rising sea levels, and high temperatures. Therefore, as far as we know, this is the first study to examine the effects of climate change on coastal hotels in Egypt and evaluated the potential vulnerability of Alexandria to the projected climate change (the destination level). We also assessed the vulnerability of Alexandria’s coastal hotels to the projected climate change (at the sector level). Thus, this study not only contributes to deepening the understanding of how coastal hotels in developing countries like Egypt adapt to climate change by examining their practices and initiatives, but also to the development of studies that focus on environmental practices in the tourism and hospitality industry. The study is also a response to Goal 13.1 of the Sustainable Development Goals, which states that all countries should be more resilient to natural disasters and climate-related risks.

Unsurprisingly, the analysis of the vulnerability of Alexandria’s coastal hotels to potential climate change highlights that more than half of the sandy beaches between Montazah and Salsilah, with a length of 14.5 km, disappeared after the widening of the Corniche Highway toward the sea, giving rise to coastal cells “starved in sediment”, resulting in the slow and continuous erosion of about 20 cm per year due to natural factors combined with a lack of sediment. The study concludes that the hotel industry in Alexandria is vulnerable to SLR based on the results of the geospatial analysis of coastal hotels. Approximately 1 in 10 of the investigated hotels is at risk of inundation, which amounts to four hotels. Any adaptation strategies should consider this dreadful scenario.

Additionally, the results revealed that Alexandria’s beachfront hotels anticipate severe financial losses due to climate change threats and are taking significant adaptation precautions. This reveals an understanding of the looming environmental risks and the exploitation of advanced technology and management as an important means to adapt to and mitigate the consequences of climate change. However, the results of this study indicated that there are two groups of hotels. The first group includes large-sized hotels (with a capacity of more than 100 rooms) belonging to the four and five-star categories and adopting environmental programs and measures in their facilities. These hotels have large and varied adaptation measures. On the other hand, small-sized hotels (<100 rooms) have provided minimal adaptation measures, reflecting the lower adaptive capacity of these hotels compared to larger hotels. Adapting to the risks of climate change is an ongoing process that requires coordination and cooperation from all decision-makers. The findings of this study are expected to help as a diagnostic tool for problem management and preventive decision making. Policymakers at all levels must include the hotel industry as a key element when formulating national climate change adaptation strategies. There is a broad spectrum of direct, indirect, and induced effects brought on by the industry. The degree to which hotels adapt to climate change will ultimately affect the economic, social, and cultural viability of the destination.

Despite the exploratory nature of the study, some recommendations can be formulated. Coastal hotels should be pertinent to anticipate future climate changes, elicit their consequences, and build more robust and sustainable adaptation strategies. In addition, the hotel’s adaptive strategies should be prioritized to prepare businesses for these impending challenges. Additionally, instead of acting individually and exploiting climate change adaptation as a competitive advantage, it would be better for hotels to work together to mitigate potential risks across Alexandria as a whole. Certainly, no hotel will thrive if potential guests fret that climate change will ruin their vacation. Large hotels with outstanding operational expertise have a responsibility to help smaller hotels adjust to the threats of climate change by sharing their own experiences. We argue that higher-level hotel operators react to climate change issues better than lower-standard hotel operators due to accessible resources such as financial and human capital. Premium and luxury hotels have superior local, regional, and worldwide networking, whereas budget and mid-scale hoteliers have limited networks. Furthermore, it is critical for the Alexandria government and local authorities to publish more and continually update the data and information regarding climate change and future projections for the city of Alexandria to facilitate timely and appropriate decision making. It is also crucial to provide substantial financial and technical assistance to small hotels with limited adaptability. The government should aid the hotel industry by passing laws, funding education initiatives, and establishing emission caps for major hotels. All hotels should, however, take the initiative to reduce their energy use and GHG emissions [90]. Drawing the topic to the forefront is crucial if we want individuals to take it seriously. Mainstream media (television and radio) and online social networks are only two of many potential avenues to raise the public awareness of climate change and other environmental challenges and foster greater environmental literacy. More than that, the government should make sure that environmental education is part of all levels of schooling.

## 6. Limitations and Future Research

The effects of climate change on vulnerable coastal regions like Alexandria are a new and contentious issue that might have far-reaching repercussions for national security. Due to these factors, data collection was protracted. Egypt-specific secondary sources are limited, muddled, and out of date. Furthermore, tracking the zone’s changes to obtain solid numbers concerning these effects takes a lot of time and resources. This highlights the importance of carrying out other adaptation studies of coastal hotels in other coastal areas, whether in Egypt or other countries in the Mediterranean region, to validate the findings. As a result, the fact that there have not been many studies about how climate change affects coastal hotels leaves a research gap for future studies. Furthermore, given the economic and social importance of tourism activities in this region, scientific research should focus on the resilient adaptive measures that can be adopted to mitigate potential risks. Because of the breadth of the study, it did not cover other aspects of the tourist industry such as attractions, modes of transportation, restaurants, and other food and beverage establishments. As important components of a coastal tourism destination, they should be incorporated into future adaptation studies. For hotels, cutting down on, or even eliminating, gas emissions might be aided by investments in new technologies and the development of technical solutions. It is crucial to upgrade to newer technology, especially in kitchens, which use a lot of energy.

## Figures and Tables

**Table 1:** Hotel climate change risks and challenges.

Challenges	Description
*Raw material supply issues*	◦ Water scarcity, poor crop yields, and low livestock output contribute to agricultural product scarcity. ◦ Low quality of raw materials due to diseases, infections, or severe weather spectacles.
*Customer behavior*	◦ Volatility in demand due to environmental awareness.
*Production relocation*	◦ Relocating hotel facilities to more robust geographical areas.
*Changes in process efficiency and effectiveness*	◦ Temperature increases may have an impact on process performance and output quality.
*Transformations in product quality*	◦ Reduced product quality as a consequence of changing seasons, resulting in lower-quality raw materials.
*Reduction in labor productivity*	◦ Heat stress and other climate-related events may reduce employee productivity.
*Infrastructural and physical assets destruction*	◦ Extreme weather conditions like floods and storms, for example, can cause damage to structures, transport facilities, utilities, and communications networks. ◦ Delays in delivering goods/services due to damage to transportation infrastructure.
*Destruction to the economy*	◦ Climate change is causing a mass emigration of people and might result in a decrease in the number of customers.
*Added costs/expenses*	◦ Increased costs due to a lack of resources. ◦ Agriculture disease and pest control costs. ◦ Costs and/or consumption of energy. ◦ Insurers’ and maintenance expenses. ◦ Cost of developing and adopting new technology and creative solutions (e.g., low-carbon technology, climate-resilient structures, and temperature control technologies).
*Financial decline*	◦ Profits and income are reduced as a result of rising commodity prices and operating expenses.
*Regulations modifications*	◦ Environmental restrictions (e.g., GHG reduction). ◦ Regulations governing disaster management.
*Negative impact on the hotel’s reputation*	◦ Customer dissatisfaction due to product quality or delivery delays.
*Changes in energy usage*	◦ Changes in energy demand and consumption.

Source: Kara et al. [48].

**Table 2:** Climate change adaptation practices by hotels.

Type of Adaption	Description
*Technical*	Using cutting-edge research and development to determine strategies for adapting to climate change and reducing exposure to risk. For example, the collection of rainwater and recycling systems.
*Managerial*	By taking new and diverse approaches to the issue, hotels can better control climate change. In addition to being susceptible to climate change’s direct effects, these factors also leave businesses open to additional threats such as shifts in available resources or a decline in patronage. Adaptations include water conservation strategies, low-season closures, and the introduction of new products and markets in response to rising summer temperatures and varying seasonality in consumer demand.
*Policies*	In terms of policies, hotels should be careful to design short-term strategies to reduce exposure to climate impacts and economic uncertainty. In the long-term, these strategies should contribute to reducing exposure to climate change and limiting the social and economic consequences. For example, coastal hotels should follow rules (like building codes) to deal with storm surges that can damage buildings and cause erosion.
*Education*	Education and increased awareness of climate change issues can reduce the external pressures on stakeholders (customers and employees). This can contribute to better adaptation and help develop appropriate control activities. For example, water conservation education.
*Behavioral*	Individual behavioral modifications include changing the behavior of tourists and restricting some malpractices; adjusting the scheduling of the visits; and change of the destination.

Source: Adapted from [8,42].

**Table 3:** The impact of sea level rise scenarios on Egypt’s northern coast.

Sea Level Rise Impact	Lowest Scenario, RCP 2.6	Moderate Scenario, RCP 4.5	Highest Scenario, RCP 8.5
Shoreline retreat (m)	23.4 to 29.0	28.2 to 35.0	39.6 to 49.1
Annual erosion rate (m/year)	0.24 to 0.30	0.29 to 0.37	0.41 to 0.51
Eroded beach area (Km^2^)	7.78	9.49	13.31
Beach loss (%)	25.57%	30.84	43.25

Source: Sharaan and Udo [68].

**Table 4:** Study’s mean SLR projections.

Scenario	Average Global Sea Level Rise (m)
RCP 8.5 (2046–2065)	0.30
RCP 8.5 (2081–2100)	0.63
SSP 5 (2150)	1.32
SLR 2m	2.0

**Table 5:** Characteristics of the respondent hotels.

Attribute	Freq. (N = 36)	%
**Hotel’s category**		
Five-star hotel (upscale)	9	25%
Four-star hotel (mid–scale)	7	19%
Three-star hotel (budget)	20	56%
**Duration of market presence**		
<3 Years	9	25%
From 3–10 Years	22	61%
>10 Years	5	14%
**The number of hotel rooms**		
<50 rooms	5	14%
From 50 to 100 rooms	15	42%
>100 rooms	16	44%
**Environmental management systems implementation status**		
Ready	6	17%
Future	26	72%
Partial	4	11%

**Table 6:** Climate change adaptation measures by coastal hotels in Alexandria.

Adaption Measures	Mean	SD
**Technical measures (TM) [Mean, 4.04]; [Cronbach’s** α**, 0.849]**		
Improving hotel building insulation and natural ventilation.	4.28	0.845
Reducing the energy consumption for cooling, heating, and ventilation.	4.00	0.900
Optimize building materials to withstand severe storms or heavy rains during winter.	3.87	0.970
Expanding the drainage systems to adapt to heavy rainfall during winter.	4.11	0.711
Planting a green belt of trees and other plants to reduce wind force.	3.90	0.661
Improving the efficiency of water use and building additional water storage capacity.	4.26	0.697
Reinforcing beaches/coastal areas using natural or artificial barriers.	4.00	1.119
Creating plans and procedures for emergency evacuation.	3.94	1.019
**Managerial measures (MM) [Mean, 3.88]; [Cronbach’s** α**, 0.855]**		
Suggesting safe activities for customers during and after impactful weather events.	3.89	0.862
Carrying out hotel operations in an environmentally compatible manner.	3.85	0.795
Establishing both immediate and long-term planning for effective adaption strategies.	3.91	0.985
**Policies (PM) [Mean, 3.99]; [Cronbach’s** α**, 0.825]**		
Complying with the national environmental laws and regulations.	4.16	0.655
Complying with environmental initiatives to protect and conserve natural resources.	3.38	1.000
Complying with the terms of environmental permits that are required for operations.	3.98	0.897
**Awareness raising measures (ARM) [Mean, 3.53]; [Cronbach’s** α**, 0.845]**		
Educating staff about potential climate change risks.	3.92	0.723
Educating staff and community about climate change implications and adaptation.	3.99	0.890
Instructing guests and staff on how to handle climate change threats.	3.28	0.774
Participating in public campaigns about climate change.	3.40	0.974
Informing all stakeholders about the hotel’s environmental performance.	3.05	0.784

**Table 7:** Climate adaptation rate based on key hotel characteristics.

Adaptation Measures	Hotel Category			Duration			Hotel Size			EMS Status		
	5-Star (*n* = 9)	4-Star (*n* = 7)	3-Star (*n* = 20)	<3 (*n* = 9)	3–10 (*n* = 22)	>10 (*n* = 5)	Small (*n* = 5)	Medium (*n* = 15)	Large (*n* = 16)	Ready (*n* = 6)	Partial (*n* = 4)	Future (*n* = 26)
*Technical measures*												
TM1	89%	71%	60%	100%	95%	40%	60%	100%	93%	100%	75%	50%
TM2	89%	85%	55%	78%	90%	60%	40%	87%	87%	83%	75%	73%
TM3	78%	57%	60%	89%	86%	40%	60%	93%	87%	83%	75%	65%
TM4	56%	43%	70%	89%	77%	40%	80%	73%	100%	67%	50%	61%
TM5	67%	57%	55%	100%	95%	60%	60%	80%	93%	67%	25%	61%
TM6	89%	71%	50%	100%	77%	20%	20%	80%	87%	83%	50%	54%
TM7	89%	71%	55%	89%	100%	40%	40%	80%	80%	83%	50%	58%
TM8	78%	86%	60%	100%	95%	20%	20%	73%	87%	67%	50%	54%
*Managerial measures*												
MM1	67%	57%	75%	78%	82%	40%	40%	80%	81%	83%	75%	61%
MM2	56%	43%	70%	89%	77%	40%	40%	80%	87%	100%	50%	65%
MM3	67%	71%	60%	89%	68%	60%	60%	80%	75%	83%	75%	65%
*Policies*												
PM1	78%	71%	60%	100%	86%	40%	20%	100%	87%	100%	75%	77%
PM2	67%	85%	55%	67%	73%	40%	20%	73%	75%	100%	100%	58%
PM3	100%	71%	13	78%	91%	20%	20%	93%	87%	83%	75%	77%
*Awareness raising measures*												
ARM1	78%	85%	75%	78%	86%	40%	40%	87%	81%	83%	50%	81%
ARM2	67%	57%	70%	89%	82%	40%	0	93%	94%	67%	50%	88%
ARM3	67%	43%	55%	55%	77%	20%	0	80%	69%	83%	75%	61%
ARM4	44%	57%	60%	67%	68%	60%	20%	87%	75%	67%	75%	65%
ARM5	78%	71%	55%	44%	68%	60%	20%	67%	69%	50%	50%	65%

## Data Availability

Not applicable.
